# 3-Hydroxybenzoate 6-Hydroxylase from *Rhodococcus jostii* RHA1 Contains a Phosphatidylinositol Cofactor

**DOI:** 10.3389/fmicb.2017.01110

**Published:** 2017-06-16

**Authors:** Stefania Montersino, Evelien te Poele, Roberto Orru, Adrie H. Westphal, Arjan Barendregt, Albert J. R. Heck, Robert van der Geize, Lubbert Dijkhuizen, Andrea Mattevi, Willem J. H. van Berkel

**Affiliations:** ^1^Laboratory of Biochemistry, Wageningen University and ResearchWageningen, Netherlands; ^2^Microbial Physiology, Groningen Biomolecular Sciences and Biotechnology Institute, University of GroningenGroningen, Netherlands; ^3^Department of Biology and Biotechnology, University of PaviaPavia, Italy; ^4^Biomolecular Mass Spectrometry and Proteomics, Bijvoet Center for Biomolecular Research and Utrecht Institute for Pharmaceutical Research, Utrecht UniversityUtrecht, Netherlands

**Keywords:** expression strain, flavoprotein, monooxygenase, phospholipid, *Rhodococcus*

## Abstract

3-Hydroxybenzoate 6-hydroxylase (3HB6H, EC 1.13.14.26) is a FAD-dependent monooxygenase involved in the catabolism of aromatic compounds in soil microorganisms. 3HB6H is unique among flavoprotein hydroxylases in that it harbors a phospholipid ligand. The purified protein obtained from expressing the gene encoding 3HB6H from *Rhodococcus jostii* RHA1 in the host *Escherichia coli* contains a mixture of phosphatidylglycerol and phosphatidylethanolamine, which are the major constituents of *E. coli’s* cytoplasmic membrane. Here, we purified 3HB6H (*Rj*HB6H) produced in the host *R. jostii* RHA#2 by employing a newly developed actinomycete expression system. Biochemical and biophysical analysis revealed that *Rj*3HB6H possesses similar catalytic and structural features as 3HB6H, but now contains phosphatidylinositol, which is a specific constituent of actinomycete membranes. Native mass spectrometry suggests that the lipid cofactor stabilizes monomer-monomer contact. Lipid analysis of 3HB6H from *Pseudomonas alcaligenes* NCIMB 9867 (*Pa*3HB6H) produced in *E. coli* supports the conclusion that 3HB6H enzymes have an intrinsic ability to bind phospholipids with different specificity, reflecting the membrane composition of their bacterial host.

## Introduction

*Rhodococcus jostii* RHA1 is a biotechnologically and environmentally important bacterium from the order Actinomycetales. Together with the genera *Nocardia, Corynebacterium* and *Mycobacterium, Rhodococcus* forms a distinct group of bacteria called mycolata (Finnerty, 1992; Brennan and Nikaido, 1995; Chun et al., 1996; Gürtler et al., 2004), characterized by a complex cell envelope (Sutcliffe, 1998; Guerin et al., 2010; De Carvalho et al., 2014) and an impressive catabolic diversity, allowing adaptation to different carbon sources for growth (van der Geize and Dijkhuizen, 2004). In comparison with other mycolata, *R. jostii* RHA1 is particularly rich in oxygenases (203 putative genes) and ligases (192 putative genes), gained primarily through ancient gene duplications or acquisitions (McLeod et al., 2006; Yam et al., 2010).

**FIGURE 1:**
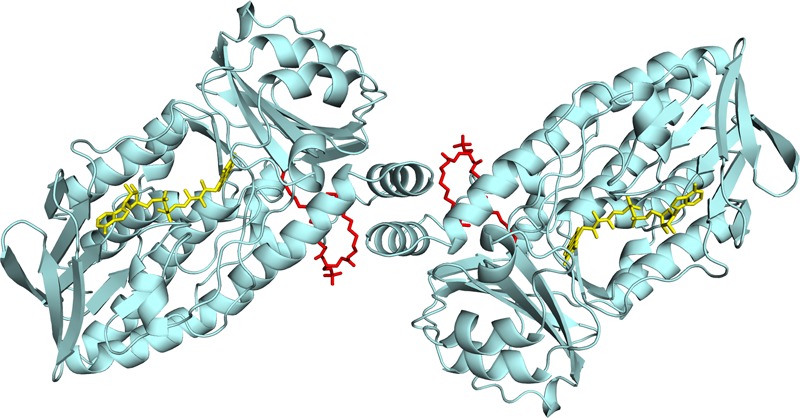
Lipid binding in 3HB6H from *R. jostii* RHA1. Cartoon of the three-dimensional structure of the 3HB6H dimer (Montersino et al., 2013). The protein is shown in light blue and the FAD cofactor is depicted as stick model in yellow. The lipid cofactor and the aromatic substrate are shown as stick models in red and dark blue, respectively.

We recently reported the crystal structure of *R. jostii* RHA1 3-hydroxybenzoate 6-hydroxylase (3HB6H), produced as a recombinant protein in *Escherichia coli* (Montersino et al., 2013). 3HB6H (EC 1.13.14.26) is a NADH and FAD-dependent monooxygenase that catalyzes the *para-*hydroxylation of 3-hydroxybenzoate to 2,5-dihydroxybenzoate, using a Tyr-His pair for substrate binding and catalysis (Sucharitakul et al., 2015). The crystal structure analysis revealed that 3HB6H has the conserved fold of group A flavoprotein hydroxylases (Montersino et al., 2011; Huijbers et al., 2014), but differs from the other family members in additional binding of phospholipids. The tightly bound phospholipids were identified as a mixture of PG and PE, which are the major constituents of the *E. coli* cytoplasmic membrane (Pulfer and Murphy, 2003; Oursel et al., 2007). The fatty acyl chains of the phospholipid ligands of 3HB6H protrude into the substrate-binding pockets, whereas the surface-exposed hydrophilic headgroups are involved in enzyme dimerization (**Figure 1**) (Montersino et al., 2013).

To shed more light on the role of these lipid guests, bearing in mind the different lipid compositions of Gram-positive and Gram-negative bacterial membranes (Finnerty, 1992; Sutcliffe, 1998), in the present work we produced *Rj*3HB6H in a newly developed *R. jostii* RHA1#2 expression strain and, in addition, 3HB6H from *Pseudomonas alcaligenes* NCIMB 9867 (*Pa*3HB6H) in *E. coli*. Biochemical and biophysical characterization revealed that *Rj*3HB6H possesses similar catalytic and structural features as 3HB6H, but contains PI as glycerophospholipid ligand. Lipid analysis of *Pa*3HB6H indicates that lipid binding is an intrinsic property of prokaryotic 3-hydroxybenzoate 6-hydroxylases.

## Materials and Methods

### Chemicals

Aromatic compounds were purchased from Sigma-Aldrich (St Louis, MO, United States) and Acros Organics (Morris Plains, NJ, United States). Catalase, FAD, FMN, arabinose, antibiotics, Terrific broth (TB) and LB broth (Miller) (LB) were from Sigma-Aldrich (St Louis, MO, United States). Pefabloc SC and DNase I were obtained from Roche Diagnostics GmbH (Mannheim, Germany). Restriction enzymes and *Pfu* DNA polymerase were from Thermo Fischer Scientific (United States). 4-androstene-3,17-dione was from Merck (Oss, Netherlands). Crystallization kits were purchased from New Hampton (Aliso Viejo, CA, United States). Immobilized metal affinity chromatography columns (His GraviTrap) were from GE Healthcare Bioscience AB (Uppsala, Sweden). All other chemicals were from commercial sources and of the purest grade available.

### Bacterial Strains and Primers

All bacterial strains and primers used in this study are listed in **Tables 1, 2**.

**Table 1:** List of primers used in PCR.

*oriT-*F	5′-CATAGTCCACGACGCCC-3′
*oriT-*R	5′-TCTTTGGCATCGTCTCTCG-3′
*egfp*-*Pci*I-F	5′-GCACATGTCGGAGGTCCATATGGCCATGGT-3′
*egfp*-*Pci*I-R	5′-GCACATGTATTACTTGTACAGCTCGTCCATGC-3′
*reg1*-*Bgl*II-F	5′-GGAGATCTGACATTCCCGCGATACG-3′
*prmA*-*Nde*I-R	5′-GGCATATGTGCGCCTCCTGGATCG-3′
*reg2*-*Pci*I-F	5′-GGACATGTCCCGGTCCTCCACCACCCCGTCT-3′
*reg2*-*Pci*I-R	5′-GGACATGTCGGTGCGGGCGACGTCATATGTCG-3′
MCS-*Nde*I-F	5′-TTGCATATGCACCGCGGTGGC-3′
MCS-*Pci*I-R	5′-GGGAACATGTGCTGGGTACC-3′

**Table 2:** List of bacterial strains.

*R. jostii* RHA1	The complete genome of *Rhodococcus* sp. RHA1 provides insights into a catabolic powerhouse. (McLeod et al., 2006)
*R. jostii* strain RHA1#2	Used as a host for protein production. This strain is a spontaneous mutant of *R. jostii* RHA1, carrying deletions of ∼0.9 Mb on the 1.12 Mb linear plasmid pRHL1 and ∼0.2 Mb on the 0.44 Mb linear plasmid pRHL2. The deletions together comprise ∼11.3% of the 9.7 Mb *R. jostii* genome.
*E. coli* DH5α	Used as host for cloning procedures.
*E. coli* TOP10	Used for production of 3HB6H (Montersino and van Berkel, 2012) and *Pa*3HB6H.

### Construction of *Rhodococcus* Expression Vector Q2+

The *E. coli-Rhodococcus* shuttle vector pRESQ (van der Geize et al., 2002) was modified by insertion of the RP4 *oriT* of pK18*mobsacB* (Schäfer et al., 1994) enabling trans-conjugal transfer of the resulting vector. For this, the *oriT*-region was amplified from pK18*mobsacB* by PCR using forward primer *oriT-*F and reverse primer *oriT-*R (**Table 1**). The obtained 549 bp PCR-product was cloned into the *Sma*I-site of pRESQ, resulting in pQmob. A duplicate region of 424 bp on pQmob was removed by deleting the 760 bp *Xba*I-*Bsp*HI fragment, yielding pQmobΔd. The *egfp* gene from pIJ8630 (Sun et al., 1999) was amplified by PCR using forward primer *egfp*-*Pci*I-F, containing a *Pci*I restriction site, and reverse primer *egfp*-*Pci*I-R, also containing a *Pci*I restriction site (**Table 1**). The 744 bp *Pci*I-*Pci*I fragment containing the *egfp* gene was cloned into the *Pci*I-site of pQmobΔd to generate peGFPQ.

The *R. jostii* strain RHA1 genomic region consisting of gene *ro00440*, its promoter region and the *prmA* promoter (P*prmA*) (here referred to as region *reg1*-P*prmA*; GenBank accession number CP000431: nt 521345 - nt 523358) was amplified from genomic DNA of *R. jostii* RHA1 by PCR using forward primer *reg1*-*Bgl*II-F, containing a *Bgl*II restriction site, and reverse primer *prmA*-*Nde*I-R, containing an *Nde*I restriction site (**Table 1**). The 2014 bp *Bgl*II-*Nde*I *reg1*-P*prmA* fragment was cloned into the *Bgl*II-*Nde*I sites of peGFPQ, yielding prMOeGFPQ1.

The *R. jostii* RHA1 gene *ro00452* and its promoter region (here referred to as region *reg2*; CP000431: nt 534363 – nt 536227) were amplified by PCR using forward primer *reg2*-*Pci*I-F, containing a *Pci*I restriction site and reverse primer *reg2*-*Pci*I-R, also containing a *Pci*I restriction site (**Table 1**). The 1880 bp *Pci*I-*Pci*I *reg2* fragment was cloned into the *Pci*I-site of prMOeGFPQ1, resulting in prMOeGFPQ2.

For construction of expression vector Q2+, the *egfp* gene of prMOeGFPQ2 was replaced with a multiple cloning site (MCS). For this, part of the MCS of pBluescript KS was amplified by PCR using forward primer MCS-*Nde*I-F, containing an *Nde*I restriction site and reverse primer MCS-*Pci*I-R, containing a *Pci*I restriction site (**Table 1**). The 125 bp *Nde*I-*Pci*I MCS fragment was cloned into the *Nde*I–*Pci*I site of prMOeGFPQ2, replacing the *Nde*I–*Pci*I region containing *egfp*, resulting in the expression vector Q2+.

### Cloning and Production of 3HB6H in *R. jostii* RHA1#2

The 1321 bp *Nde*I-*Xmn*I fragment of pBAD-3HB6H-His_6_ (Montersino and van Berkel, 2012) containing the 3HB6H gene including the His_6_-tag, was cloned into the *Nde*I-*Hind*III (Klenow-fragment treated) site of expression vector Q2+ to generate Q2+-3HB6H-His_6_.

*Rhodococcus* cells were electroporated as described previously (van der Geize et al., 2000). Prior to electroporation, plasmid DNA was desalted by dialyzing 10 μL plasmid DNA for 30 min on a Millipore “V” Series filter disk (0.025 μm) floating on MiliQ water.

Cultures of *R. jostii* RHA1#2 were grown in LB broth supplemented with 50 μg⋅mL^-1^ kanamycin at 30°C at 200 rpm. *R. jostii* RHA1#2 cells harboring Q2+3HB6H-His_6_ were grown overnight in 3 mL LB broth, diluted 1:300 in 300 mL LB broth in a 3 L Erlenmeyer flask and grown for 20–24 h. Cultures were induced by adding 2 mM 4-androstene-3,17-dione dissolved in acetone. Growth was continued for 48 h after induction. Cells were harvested by centrifugation at 4°C and pellets were washed once with ice-cold 20 mM potassium phosphate, pH 7.2, containing 300 mM NaCl. After centrifugation at 4°C, cells were stored at -20°C.

### Cloning and Production of 3HB6H from *Pseudomonas alcaligenes* NCIMB 9867

The *xlnD* gene sequence encoding for *Pa*3HB6H (UniProt: Q9F131) was synthesized and subcloned in a pBAD vector by GeneArt (Invitrogen, Carlsbad, CA, United States). The resulting construct (pBAD-*Pa*3HB6H-His_6_) was verified by automated sequencing of both strands and electroporated into *E. coli* TOP10 cells for recombinant expression.

For enzyme production, *E. coli* TOP10 cells, harboring the pBAD-*Pa*3HB6H-His_6_ plasmid, were grown in TB medium at 37°C supplemented with 100 μg⋅mL^-1^ ampicillin until an optical density (OD_600__nm_) of 0.8 was reached. Expression was induced by the addition of 0.02% (w/v) arabinose and incubation was continued for 40 h at 17°C. Cells were harvested by centrifugation at 4°C and stored at -20°C.

### Enzyme Purification

*Rj*3HB6H was purified to apparent homogeneity using an Åkta Explorer chromatography system (GE-Healthcare). *R. jostii* RHA1#2 cells containing the recombinant protein were suspended in ice-cold 20 mM potassium phosphate, pH 7.2, containing 300 mM NaCl, 1 mM Pefabloc SC, 1 mg DNAse and 100 μM MgCl_2_, and subsequently passed twice through a precooled French Pressure cell (SLM Aminco, SLM Instruments, Urbana, IL, United States) at 16,000 psi. The resulting homogenate was centrifuged at 25,000 × *g* for 45 min at 4°C to remove cell debris, and the supernatant was applied onto a Ni-NTA agarose column (16 mm × 50 mm) equilibrated with 20 mM potassium phosphate, pH 7.2, containing 300 mM NaCl. After washing with five volumes of equilibration buffer, the enzyme was eluted with 300 mM imidazole in equilibration buffer. The resulting *Rj*3HB6H fraction was supplemented with 100 μM FAD and loaded onto a Source Q-15 anion exchange column (16 mm × 90 mm), pre-equilibrated with 50 mM Bis-Tris, 0.1 mM EDTA, pH 7.2. After washing with two volumes of equilibrating buffer, the enzyme was eluted with a linear gradient of 0–1 M NaCl in the same buffer. Active fractions were pooled, concentrated to 10 mg⋅mL^-1^ using ultrafiltration (Amicon 30 kDa cutoff filter), and applied onto a Superdex S-200 (26 mm × 600 mm) column running in 50 mM potassium phosphate, 150 mM NaCl, pH 7.2. Active fractions were concentrated to 10 mg⋅mL^-1^ using ultrafiltration (Amicon 30 kDa cutoff filter) and dialyzed at 4°C against 50 mM Bis-Tris, pH 7.2. The final *Rj*3HB6H preparation showed a single band after SDS-PAGE. The specific activity of the purified enzyme was 21 U mg^-1^ using the standard activity assay (**Table 3A**).

**Table 3A:** Purification of *Rj*3HB6H produced in *R. jostii* RHA1#2.

Step	Protein (mg)	Activity (U)	Specific activity (U⋅mg^-1^)	Yield (%)
Cell extract	208	355	2	100
His GraviTrap	37	300	8	84
Mono-Q	16	230	14	65
His GraviTrap	7	168	21	47

*Pa*3HB6H was purified to apparent homogeneity, applying essentially the same procedure as described above for *Rj*3HB6H. The final *Pa*3HB6H preparation showed a single band after SDS-PAGE. The specific activity of the purified enzyme was 34 U⋅mg^-1^ using the standard activity assay (**Table 3B**).

**Table 3B:** Purification of *Pa*3HB6H produced in *E. coli.*

Step	Protein (mg)	Activity (U)	Specific activity (U⋅mg^-1^)	Yield (%)
Cell extract	1080	260	0.2	100
His GraviTrap	45	244	5	94
Mono-Q	12	225	19	87
His GraviTrap	5	170	34	66

Purified enzymes were flash frozen in 1 mL aliquots in liquid nitrogen and stored at -80°C. Before use, thawed enzyme samples were incubated with 0.1 mM FAD and excess FAD was removed using a gel filtration column (10 mm × 100 mm) containing Bio-Gel P-6DG.

### Biochemical Characterization

Molar absorption coefficients of protein-bound FAD were determined from absorption spectra of *Rj*3HB6H and *Pa*3HB6H recorded in the presence and absence of 0.1% (w/v) SDS, assuming a molar absorption coefficient for free FAD of 11.3 mM^-1^⋅cm^-1^ at 450 nm. The enzyme concentration of *Rj*3HB6H was determined by measuring the absorbance at 453 nm using a molar absorption coefficient for protein-bound FAD of 10.3 mM^-1^⋅cm^-1^. The enzyme concentration of *Pa*3HB6H was determined by measuring the absorbance at 450 nm using a molar absorption coefficient for protein-bound FAD of 11.0 mM^-1^⋅cm^-1^. *Rj*3HB6H and *Pa*3HB6H activity was determined at 25°C by measuring NADH consumption at 360 nm (Montersino and van Berkel, 2012). The standard assay mixture contained 50 mM Tris-SO_4_, pH 8.0, 200 μM 4-hydroxybenzoate and 250 μM NADH. Steady-state kinetic parameters were determined from measurements at 25°C in 50 mM Tris-SO_4_, pH 8.0. Hydroxylation efficiencies were determined by oxygen consumption experiments, essentially as described before (Montersino and van Berkel, 2012).

### Crystallization and Structure Determination

Crystals of *Rj*3HB6H for structure determination were obtained by the sitting drop vapor diffusion method at 20°C by mixing equal volumes (2 μL) of protein and reservoir solutions. Protein solutions consisted of 30 mg⋅mL^-1^ enzyme in 1 mM FAD, 2 mM 3-hydroxybenzoate, and 50 mM Bis-Tris, pH 7.2, whereas precipitant solutions consisted of 30% PEG 4000, 0.2 M lithium sulfate, and 0.1 M Tris-HCl, pH 8.5. Yellow crystals grew in 1 day.

X-ray diffraction data were collected at Grenoble and processed with the CCP4 package (Winn et al., 2011). The *Rj*3HB6H structure was solved by molecular replacement using the structure of a monomer of 3HB6H (pdb entry: 4BJZ) as search model. Crystallographic computing and model analysis were performed with COOT (Emsley et al., 2010), PHENIX (Adams et al., 2010) and the CCP4 package (Potterton et al., 2004). Pictures were generated with Pymol (Schrodinger, 2015) and CCP4 (Potterton et al., 2004). Data collection parameters and refinement statistics are presented in **Table 4**.

**Table 4:** Crystallographic data collection and refinement statistics of *Rj*3HB6H.

Protein Data Bank Code	5HYM
Unit cell (Å)	*a* = *b* = 106.98 *c* = 143.39
Space group	*I4_1_22*
Resolution (Å)	2.30
*R*_sym_^a,b^ (%)	15.1 (50)
Completeness*^b^* (%)	99.7 (100)
Unique reflections	18,766
Redundancy*^b^*	7.5 (5.8)
*I*/σ^b^	8.4 (3.0)
No. of atoms	3,198
Average B value (Å^2^)	33.4
*R*_cryst_^c^ (%)	20.6
*R*_free_^c^ (%)	26.2
r.m.s. bond length (Å)	0.015
r.m.s. bond angles (°)	1.75

*^a^R_*sym*_ = Σ|*I_*i*_*–*I*| /Σ*I_*i*_* where *I_*i*_* is the intensity of *i*th observation and *I* is the mean intensity of the reflection. *^b^*Values in parentheses are for reflections in the highest resolution shell. *^c^R*_*cryst*_ = Σ|*F*_*obs*__-_*F*_*calc*_| /Σ*F*_*obs*_ where *F*_*obs*_ and *F*_*calc*_ are the observed and calculated structure factor amplitudes, respectively. *R*_*cryst*_ and *R*_*free*_ were calculated using the working and test set, respectively. r.m.s., root mean square*

The atomic coordinates and structure factors of *Rj*3HB6H (code 5HYM) have been deposited in the Protein Data Bank^1^.

### Lipid Identification and Native ESI-MS Experiments

Extraction and identification of protein-bound lipids from *Rj*3HB6H and *Pa*3HB6H was performed as described for 3HB6H (Montersino et al., 2013). For nanoflow ESI-MS analysis under native conditions, enzyme samples were prepared in 50 mM ammonium acetate, pH 6.8. For analysis under denaturing conditions, enzyme samples were diluted either in 50% acetonitrile with 0.2% formic acid or in 5% formic acid. Native MS analysis was performed using a LC-T nanoflow ESI orthogonal TOF mass spectrometer (Micromass, Manchester, United Kingdom) in positive ion mode with a capillary voltage of 1.3 kV. The cone voltage was varied between 90 and 150 V and source pressure was set to 6.9 mbar to enhance transmission of large ions. Lipid identification was performed using a Quattro Ultima nanoflow triple quadrupole mass spectrometer (Micromass, Manchester, United Kingdom) in negative ion mode, with a capillary voltage of 1.3 kV and a cone voltage of 150 V. For MS/MS analysis, argon was supplied in the collision cell (2.0 × 10^-3^ bar). Collision energy was adjusted to gain optimal fragmentation. Both mass spectrometers were equipped with a Z-spray nano-electrospray ionization source. Measurements were performed by using gold-coated needles, made from borosilicate glass capillaries (Kwik-Fill; World precision Instruments, Sarasota) on a P-97 puller (from Sutter Instruments, Novato, CA, United States). Needles were coated with a gold layer using an Edwards Scancoat six Pirani 501 sputter coater (Edwards laboratories, Milpitas, CA, United States). All TOF spectra were mass calibrated by using an aqueous solution of cesium iodide (25 mg⋅mL^-1^).

### Sequence Comparison

Protein sequences were retrieved using protein resources from the National Centre for Biotechnology Information^2^ and UniProt Database^3^. Multiple sequence alignments were made using CLUSTALW (Thompson et al., 1994). Phylogenetic plots were made using FigTree^4^.

## Results

### Biochemical Properties of *Rj*3HB6H

Expression of the 3HB6H gene from *R. jostii* RHA1#2 yielded about 7 mg of purified *Rj*3HB6H protein from 10 g wet cells (**Table 3A**). *Rj*3HB6H displayed the same absorption spectrum as 3HB6H, with maxima at 274, 383, and 453 nm and a shoulder at 480 nm (Montersino and van Berkel, 2012). A molar absorption coefficient of protein-bound flavin, 𝜀_453_ = 10.3 mM^-1^ cm^-1^, was used for both proteins.

Determination of steady-state kinetic parameters revealed that *Rj*3HB6H behaves similarly as 3HB6H using 3-hydroxybenzoate as variable substrate and fixed NADH concentration (*k*_cat_ = (20 ± 1) s^-1^; *K*_M_ = (35 ± 3) μM; *k*_cat_/*K*_M_ = (5.7 ± 0.8) × 10^5^ s^-1^⋅M^-1^) and with variable concentration of NADH (preferred coenzyme) and fixed 3-hydroxybenzoate concentration (*k*_cat_ = (20 ± 1) s^-1^; *K*_M_ = (68 ± 5) μM; *k*_cat_/*K*_M_ = (3.0 ± 0.4) × 10^5^ s^-1^⋅M^-1^). *Rj*3HB6H displays a very low NADH oxidase activity (<1 U⋅mg^-1^). Uncoupling of hydroxylation of 3-hydroxybenzoate occurs to a minor extent (less than 10%), while 2,5-dihydroxybenzoate is a strong non-substrate effector (*k*_cat_ = (6 ± 0.8) s^-1^; *K*_M_ = (150 ± 30) μM; *k*_cat_/*K*_M_ = (4.0 ± 1.3) × 10^4^ s^-1^⋅M^-1^), efficiently stimulating the rate of flavin reduction by NADH (Montersino and van Berkel, 2012; Sucharitakul et al., 2012, 2013; Ni et al., 2016).

### Structural Characterization

**FIGURE 2:**
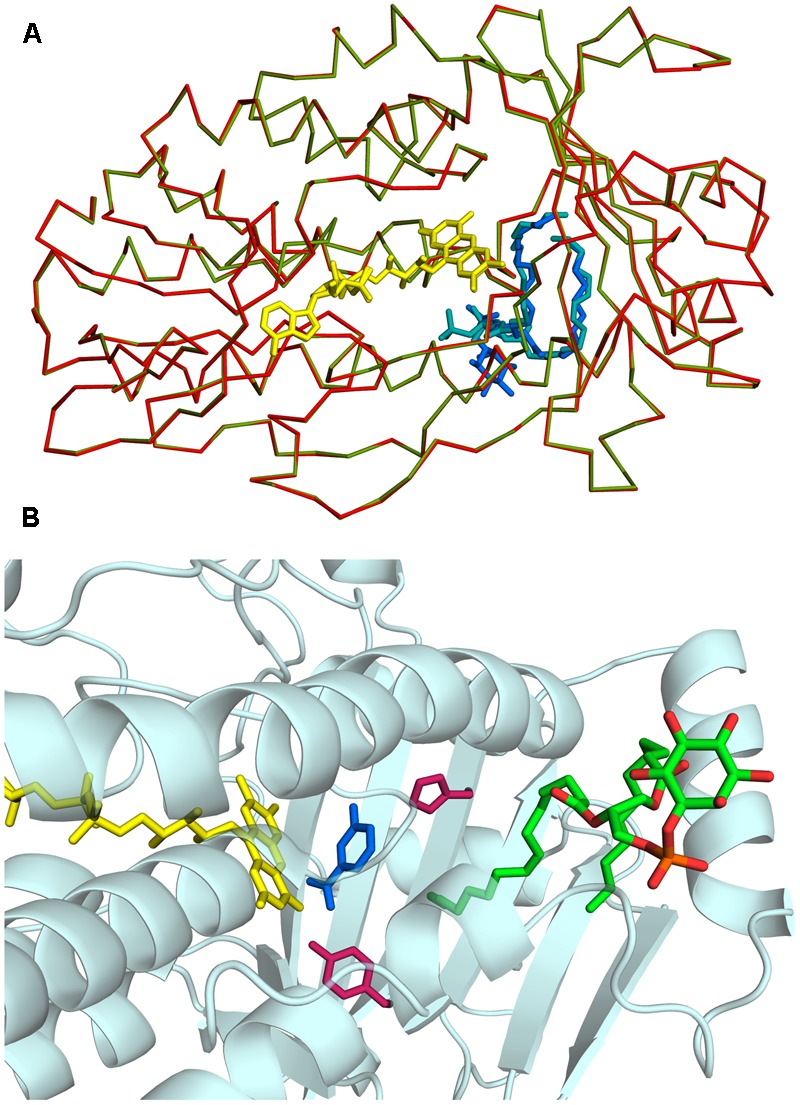
Three-dimensional structure of *Rj*3HB6H. **(A)** View of α-carbon traces of the refined structures of 3HB6H (green; pdb code 4BJZ) and *Rj*3HB6H (red; pdb code 5HYM), following superposition of corresponding main chain atoms. FAD is shown as a stick model in yellow. The lipid ligands are shown as stick models in shades of blue in the back of the protein. **(B)** The phospholipid is located in a tunnel, which runs from the dimer interface to the active site. The FAD cofactor is depicted in yellow. The lipid cofactor is colored by elements, and the active site residues His213 and Tyr217 are in dark red. The 3-hydroxybenzoate in blue is a superposition from the structure of the 3HB6H variant H213S, which contains bound substrate (pdb code 4bk1).

**FIGURE 3:**
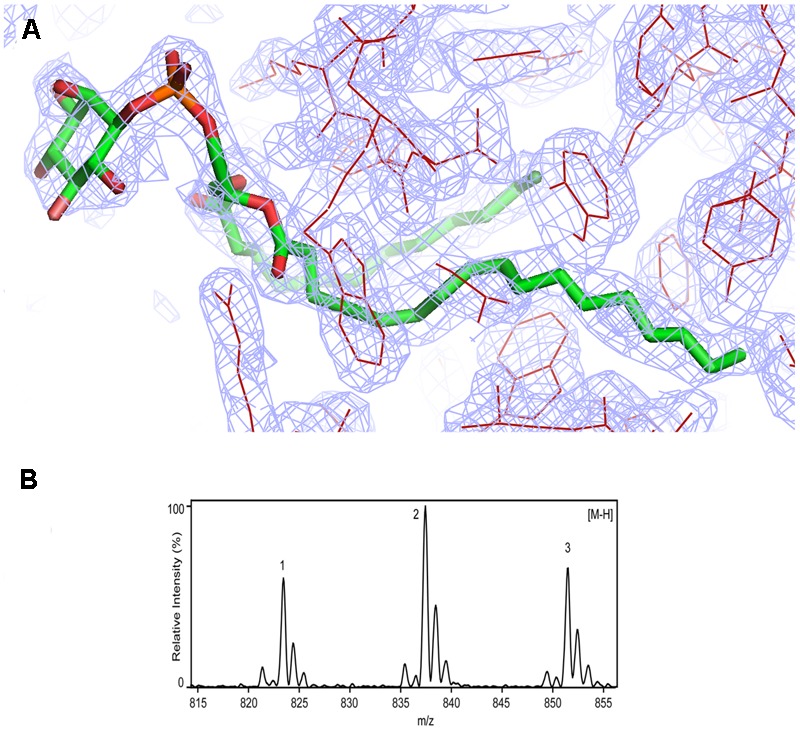
Identification of phosphatidylinositol in *Rj*3HB6H. **(A)** Weighted (2Fo-Fc) electron-density map of the lipid cofactor. **(B)** ESI-MS spectrum of lipid extract collected in negative mode. For peak assignment, see “Results” section.

*Rj*3HB6H crystals grew in similar conditions as found for 3HB6H, and are isomorphous to those of 3HB6H, where lithium sulfate was present instead of sodium acetate. The three-dimensional structure of *Rj*3HB6H was solved at 2.3 Å resolution by molecular replacement (**Table 4**). The isoalloxazine moiety of FAD was refined with full occupancy in the *in* conformation. Similar to the crystallographic analysis of 3HB6H, no substrate could be detected in the active site of the enzyme, despite presence of excess 3-hydroxybenzoate in the crystallization drop. The protein crystallizes as a dimer, just as 3HB6H (Montersino et al., 2013), and contains a phospholipid molecule in each subunit. The electron density of the phospholipid in the crystal structure was refined as two acyl chains, one of twelve and one of seventeen carbon units. Superimposition of the *Rj*3HB6H and 3HB6H models (root mean square deviation = 0.22 Å) shows minor deviations (**Figure 2A**). The phospholipid is located in a tunnel, which runs from the dimer interface to the active site (**Figure 2B**), and interacts with the opposite monomer. The phosphate group resides at the protein surface near Arg350 and Lys385, and the electron density of the headgroup is consistent with the presence of a cyclohexanehexol moiety (**Figure 3A**).

### Identification of Protein-Bound Lipid Molecules

Assignment of protein-bound phospholipids was achieved by ESI-MS analysis of the low molecular weight components extracted from denatured *Rj*3HB6H. The mass spectrum in negative mode (**Figure 3B**) displayed three main peaks with *m/z* values of 823, 837, and 851. From the MS pattern it was evident that *Rj*3HB6H binds a phospholipid with a bigger headgroup compared to that of the lipid found in 3HB6H.

Fragmentation analysis and comparison of data to reference lipid MS spectra led to a match of the obtained mass peaks with those of PI, having aliphatic chains containing 15 to 19 carbons. Peak 1 (*m/z* 823) is assigned to PI (15:0/18:0), peak 2 (*m/z* 837) is assigned to PI (16:0/18:0) (Sharp et al., 2007; Morita et al., 2011) and peak 3 ((*m/z* 851) is assigned to PI (16:0/19:0) with alternate acylate form (tuberculostearic acid) (Drage et al., 2010).

Typical fragmentation of PI was visible in the MS/MS spectra by signature peaks with *m/z* values of 153, 223, 241, and 297, representing glycerol phosphate water (*m/z* 153) and inositol headgroup fragments (Lang and Philp, 1998; Pulfer and Murphy, 2003; Oursel et al., 2007) (data not shown). Minor peaks at approximately 2 *m/z* values lower than the identified peaks represent the same PI, containing one unsaturated bond.

### Protein Oligomeric Composition

To gain further insight into the enzyme–lipid interaction, we determined the oligomeric protein composition of 3HB6H and *Rj*3HB6H using native MS (Leney and Heck, 2017). As a first step, we determined the experimental masses of the denatured proteins. The measured values (46,766 ± 4 Da for 3HB6H and 46,761 ± 2 Da for *Rj*3HB6H) agree with the mass deduced from the primary sequence, lacking the N-terminal methionine.

**FIGURE 4:**
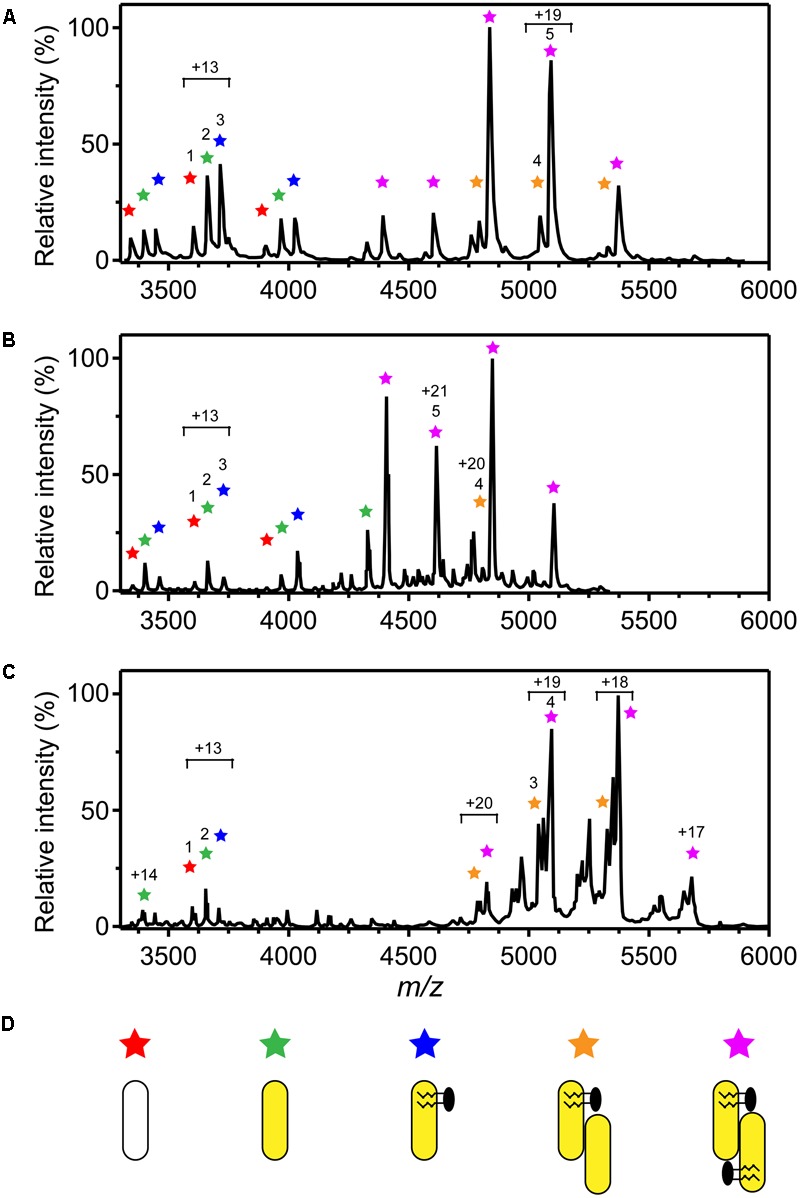
Oligomer distribution and lipid composition of 3HB6H enzymes as determined by native ESI-MS. Mass spectra were recorded in 50 mM ammonium acetate, pH 6.8. **(A)** Mass spectrum of 3HB6H. **(B)** Mass spectrum of *Rj*3HB6H. **(C)** Mass spectrum of *Pa*3HB6H. Masses and intensities of numbered peaks are listed in **Table 5**. **(D)** Cartoons of the various subunit compositions. Apoprotein is indicated in white, holoprotein in yellow and lipid molecules in black.

Native MS of 3HB6H showed eight charge states corresponding to five different protein forms (**Figure 4A** and **Table 5**). The charge state distribution ions +12, +13, and +14 represent the monomeric apoprotein (average mass 46,835 ± 5 Da; red stars), the monomeric holoprotein (average mass 47603 ± 6 Da; green stars), and the monomeric holoprotein containing additionally one PG/PE molecule (average mass 48,312 ± 7 Da; blue stars). The charge state distribution ions +18, +19, +20, +21, and +22 predominantly represent the dimeric holoprotein with either one or two PG/PE molecules bound (average mass 95,868 ± 16 Da; orange stars, and 96,643 ± 14 Da; purple stars, respectively). Tandem MS experiments revealed that one, two, three, or four ligands can be expelled from 3HB6H. The assignment of bound ligands was made on the basis of total mass increase and comparison with the mass of the native apoprotein (**Table 5**).

**Table 5:** Oligomeric forms of 3HB6H determined by native ESI-MS.

Peak^a^	*m/z*	Average mass (Da)	Δ mass (Da)
**3HB6H**			
1	3,603	46,835 ± 5	
2	3,663	47,603 ± 6	767^b^
3	3,717	48,312 ± 7	1,477^b^
4	5,048	95,868 ± 16	2,198^c^
5	5,088	96,643 ± 14	2,973^c^
***Rj*3HB6H**			
1	3,608	46,829 ± 1	
2	3,663	47,613 ± 1	784^b^
3	3,728	48,458 ± 2	1,629^b^
4	4,807	96,128 ± 5	2,470^c^
5	4,616	96,938 ± 32	3,280^c^
***Pa*3HB6H**			
1	3,651	47,448 ± 2	790^b^
2	3,706	48,167 ± 2	1,509^b^
3	5,035	95,662 ± 7	2,346^c^
4	5,073	96,738 ± 8	3,062^c^

*^a^Peaks are indicated in **Figure 4**. ^b^Calculated using apo-monomer. ^c^Calculated using apo-dimer.*

Native MS of *Rj*3HB6H also showed a range of charge state distributions (**Figure 4B** and **Table 5**). The charge state distribution ions +12, +13, and +14 represent the monomeric apoprotein (average mass 46,829 ± 1 Da; red star), the monomeric holoprotein (average mass 47613 ± 1 Da; green stars), and the monomeric holoprotein containing one PI molecule (average mass 48,458 ± 2 Da; blue star). The latter species differs from the related 3HB6H species (**Figure 4A**, blue stars) because it has a bigger lipid headgroup. The major species in the native mass spectrum of *Rj*3HB6H corresponds to the holo-*Rj*3HB6H dimer with PI bound to both subunits (average mass 96,938 ± 32 Da; **Figure 4B**, yellow stars). Only by magnification it is possible to detect a minor peak representing the holo-*Rj*3HB6H dimer with one PI bound (average mass 96,128 ± 5 Da; **Figure 4B**, orange stars). A cartoon of the different subunit compositions of 3HB6H is presented in **Figure 4D**.

### Conservation of Lipid Binding Site

To analyze whether the lipid-binding site of 3HB6H is conserved among species, we explored the natural diversity of 3HB6H enzymes. 3HB6H activity has been reported for Gram-positive and Gram-negative bacteria and for yeasts. Besides from the *R. jostii* prototype, the enzymes from *Klebsiella pneumonia* M5a1 (Suárez et al., 1995; Liu et al., 2005), *Pseudomonas alcaligenes* NCIMB 9867 (Gao et al., 2005), *Polaromonas naphthalenivorans* CJ2 (Park et al., 2007), *Corynebacterium glutamicum* ATCC 12032 (Yang et al., 2010), *Rhodococcus sp.* NCIMB 12038 (Liu et al., 2011) and *Candida parapsilosis* (Holesova et al., 2011) have been characterized to some extent.

**FIGURE 5:**
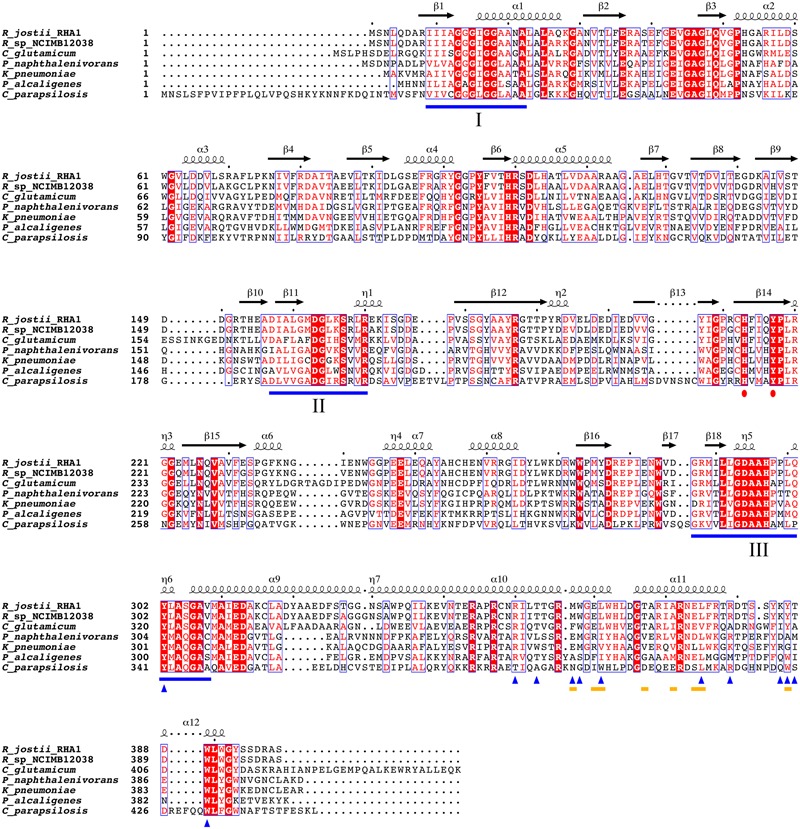
Multiple sequence alignment of known 3HB6H enzymes. UniProt ID numbers: Q0SFK6, *R. jostii* RHA1; E7CYP8, *Rhodococcus sp.* NCIMB 12038; Q8NLB6, *C. glutamicum* ATCC 12032; Q3S4B7, *P. naphthalenivorans* CJ2; Q6EXK1, *K. pneumonia* M5a1; Q9F131, *P. alcaligenes* NCIMB 9867; CPAG_03410, *C. parapsilosis*. Identical residues are shown in red. Flavin binding motifs are underlined in blue [**I**: GXGXXG; **II**: DG; **III**: GD (Eppink et al., 1997)]. The His-Tyr pair involved in substrate binding and hydroxylation is marked with red dots. The yellow lines mark residues involved in dimerization contacts. Blue triangles indicate residues involved in lipid binding. Secondary structure assigned from the 3HB6H crystal structure (4BK1). Diagram was produced using ESPript (Robert and Gouet, 2014).

From the structural data of the *R. jostii* 3HB6H enzyme and the multiple sequence alignment presented in **Figure 5** it can be inferred that residues directly involved in lipid binding in *Rj*3HB6H are not always conserved in the orthologs; among the bacterial enzymes studied, most sequence divergence occurs in 3HB6H from *P. alcaligenes* NCIMB 9867 (*Pa*3HB6H). This prompted us to study the lipid binding properties of the *Pseudomonas* 3HB6H enzyme.

Expression of the *Pa*3HB6H gene in *E. coli* TOP10 cells yielded about 10 mg of enzyme from a 1 L batch culture. Purified *Pa*3HB6H had a specific activity of 34 U⋅mg^-1^ (**Table 3B**) and migrated in SDS-PAGE as a single band with an apparent subunit mass of 47 kDa (not shown). ESI-MS established that native *Pa*3HB6H is a dimer, and not a trimer as suggested earlier (Gao et al., 2005), and that the enzyme indeed contains lipids (**Figure 4C** and **Table 5**). The mass spectrum of extracted lipids showed peaks with *m/z* values characteristic of PG and PE with aliphatic chains ranging from 14 to 19 carbons, similar to the previously identified lipids in 3HB6H from *R. jostii* RHA1 produced in *E. coli* (Montersino et al., 2013).

## Discussion

3HB6H is a flavoenzyme that catalyzes the *para*-hydroxylation of 3-hydroxybenzoate to gentisate, a key step in the catabolism of lignin-derived aromatic compounds in the soil (Pérez-Pantoja et al., 2010). Up to now, 3HB6H is the only flavoprotein monooxygenase that has been found to bind phospholipids (Montersino et al., 2013). Structural analysis showed that the hydrophobic tails of the phospholipids deeply penetrate into the substrate-binding domains, whereas the hydrophilic parts are exposed on the protein surface, connecting the dimerization domains (**Figure 1**). Attempts to obtain native lipid-free protein were not successful, indicating that the phospholipids are important to attain a properly folded protein (Montersino et al., 2013).

3HB6H binds a mixture of PG and PE, the major constituents of the *E. coli* inner membrane (Montersino et al., 2013). By expressing its gene in *R. jostii* RHA1#2, we aimed at unraveling the lipid binding abilities of 3HB6H in the original host. Although *E. coli* gives considerable higher yields (Montersino and van Berkel, 2012), significant quantities of soluble His-tagged *Rj*3HB6H were obtained. The difference in enzyme yield could be linked to the type of induction and promoter strength used in the *R. jostii* RHA1#2 strain, which is based on the propane monooxygenase operon (Sharp et al., 2007). Nevertheless, our results show that the newly developed *R. jostii* RHA1#2 strain opens new prospects for actinomycetes as host cells for production of recombinant proteins (Nakashima et al., 2005).

*Rj*3HB6H displayed similar catalytic and structural properties as 3HB6H, and the mode of lipid binding was highly conserved (**Figure 2**). Gratifyingly, the crystallographic data and mass spectrometry analysis provided clear evidence that *Rj*3HB6H contains PI as natural glycerophospholipid cofactor (**Figure 3**). The crystal structure showed that the inositol headgroups of the phospholipids are located at the protein surface, and that the *sn*-2 acyl moieties are in contact with helix 11 of the other subunit (**Figure 1**). Based on MS/MS analysis, we identified the bound phospholipids as a mixture of PIs with carbon chains between 15 and 19 carbons. One of the extracted lipids was identified as tuberculostearic acid, an alternative acylated form of palmitate present in the membranes of *Rhodococcus* and *Mycobacterium* (Drage et al., 2010).

*Rj*3HB6H is a dimer both in solution and in crystal form, but native MS showed a ratio of monomer to dimer of about 1:3 (**Figure 4B**). Release of only one PI from the dimer resulted in monomerization in the gas phase. A similar observation was made with 3HB6H, but with this enzyme more dimers containing only one bound lipid (PG or PE) were detected (**Figure 4A**). 3HB6H dimers containing two phospholipids seem to be more stable in the gas phase than dimers containing one phospholipid. This strongly supports that lipid binding near the dimer interface stabilizes monomer contacts.

Native MS-analysis showed that *Pa*3HB6H is a homodimer and not a trimer as postulated earlier (Gao et al., 2005). The dimeric nature is in agreement with the structural properties of 3HB6H and *Rj*3HB6H. MS-analysis also revealed that recombinant *Pa*3HB6H binds the same type of phospholipids as 3HB6H. This supports that lipid binding is an intrinsic property of 3HB6Hs. As a main result, it appears that the 3HB6H family uses phospholipids as a common tool to increase their dimerization strength. Phospholipid binding is independent of the type of lipid headgroup, but relies on the presence of hydrophobic tunnels running from the protein surface to the active site.

Like PG/PE in *E. coli* (Oursel et al., 2007), PI is the major lipid membrane component in *Rhodococcus* (Nigou et al., 2003). This may explain why PG/PE are found as lipid ligands of 3HB6H and *Pa*3HB6H, while PI is found in *Rj*3HB6H. PI is the precursor for lipoarabinomannan and PI-mannoside synthesis. Glycolipid synthesis and reorganization of membrane composition allow *Rhodococcus* to adapt to environmental changes (Lang and Philp, 1998; Sharp et al., 2007; Guerin et al., 2010; Morita et al., 2011; De Carvalho et al., 2014). Binding of PI may localize 3HB6H at the cytoplasmic membrane, via inositol recognition of other proteins or specific phospholipid patching on the inner side of the membrane (Morita et al., 2011). At those specific spots, uptake of aromatic compounds from the environment may be coupled more efficiently to their catabolism.

Taking together, phospholipids do not have a direct catalytic role in 3HB6H, but are involved in stabilizing the dimer contact and, possibly, substrate orientation (Montersino et al., 2013). At this stage, we cannot exclude that bound phospholipids have some other function, for instance in directing the cytoplasmic membrane localization or in guiding/protecting molecules from entering the active site. In addition, the *R. jostii* RHA1#2 expression strain described here represents a useful alternative for the production of (whole-cell) biocatalysts.

## Author Contributions

WvB and LD initiated the project; SM, EtP, AW, AB, AH, RvG, LD, AM, and WvB designed experiments and analyzed data; EtP constructed *Rhodococcus* expression vector Q2+; RO crystallized *Rj*3HB6H and determined the crystal structure; SM, AW, and AB performed analytical and biochemical experiments; SM, EtP, AM, AW, and WvB wrote the manuscript.

## Conflict of Interest Statement

The authors declare that the research was conducted in the absence of any commercial or financial relationships that could be construed as a potential conflict of interest.

## Footnotes

1 http://pdbe.org/5HYM

2 www.ncbi.nlm.nih.gov

3 http://www.uniprot.orgwww.uniprot.org

4 tree.bio.ed.ac.uk

## Abbreviations

3HB6H: recombinant 3-hydroxybenzoate 6-hydroxylase from *Rhodococcus jostii* RHA1 produced in *Escherichia coli*
*Pa*3HB6H: recombinant 3-hydroxybenzoate 6-hydroxylase from *Pseudomonas alcaligenes* NCIMB 9867 produced in *Escherichia coli*
PE: phosphatidylethanolamine
PG: phosphatidylglycerol
PI: phosphatidylinositol
*Rj*3HB6H: recombinant 3-hydroxybenzoate 6-hydroxylase from *Rhodococcus jostii* RHA1 produced in *Rhodococcus jostii* RHA1#2
